# Diastereoselective and enantioselective conjugate addition reactions utilizing α,β-unsaturated amides and lactams

**DOI:** 10.3762/bjoc.11.60

**Published:** 2015-04-23

**Authors:** Katherine M Byrd

**Affiliations:** 1 Department of Medicinal Chemistry, University of Kansas, 1251 Wescoe Hall Drive Lawrence, Kansas 66045-7582, USA

**Keywords:** α,β-unsaturated amides, α,β-unsaturated lactams, conjugate addition, Michael addition

## Abstract

The conjugate addition reaction has been a useful tool in the formation of carbon–carbon bonds. The utility of this reaction has been demonstrated in the synthesis of many natural products, materials, and pharmacological agents. In the last three decades, there has been a significant increase in the development of asymmetric variants of this reaction. Unfortunately, conjugate addition reactions using α,β-unsaturated amides and lactams remain underdeveloped due to their inherently low reactivity. This review highlights the work that has been done on both diastereoselective and enantioselective conjugate addition reactions utilizing α,β-unsaturated amides and lactams.

## Introduction

**Scheme 1:**
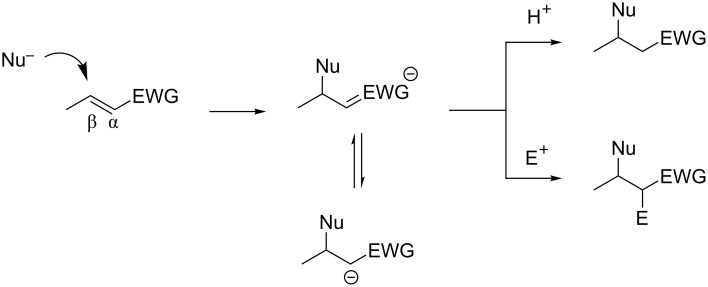
Generic mechanism for the conjugate addition reaction.

Conjugate (or 1,4- or Michael) additions [1–5] are some of the most powerful carbon–carbon bond forming reactions in the synthetic chemist’s toolbox. These reactions have been the key step in the syntheses of numerous natural products and pharmaceutically relevant compounds [6–11]. A conjugate addition (CA) reaction involves the addition of a nucleophile to an electron-deficient double or triple bond (Scheme 1). The addition takes place at the carbon that is β to the electron-withdrawing group (EWG), resulting in the formation of a stabilized carbanion intermediate. At this point, the carbanion can be either protonated to form the β-substituted product or an electrophile can be added to form the α,β-disubstituted product. The latter operation is often referred to as a three-component coupling due to the combination of the original unsaturated compound, the nucleophile, and the electrophile.

In 1883, Komnenos reported the first conjugate addition where he added the diethyl malonate anion to ethylidene malonate [12]. This reaction was not fully investigated until 1887, when Michael thoroughly studied this reaction using various stabilized nucleophiles and α,β-unsaturated systems [13]. Most of the early work, including Michael’s, involved the use of stabilized or “soft” [14] nucleophiles such as malonates and nitroalkanes. Mechanistically, one can imagine a 1,2-addition of the nucleophile occurring versus the 1,4-addition if the EWG is a carbonyl compound, but this is not observed when malonates are used as nucleophiles. It has now been well established that “soft” nucleophiles prefer a 1,4-attack whereas “hard” nucleophiles such as organomagnesium and lithium reagents prefer a 1,2-attack.

Within the past couple of decades, there has been a focus on the development of asymmetric variants of the Michael reaction. In early studies, chemists relied on chiral auxiliaries [5], heterocuprates [15–16], or natural product-based [17] ligands to induce high to modest stereoselectivity. In 1991, Alexandre Alexakis and co-workers reported the first asymmetric conjugate addition reactions where they employed organocopper reagents and chiral phosphorus-based ligands [18]. This breakthrough in the field led to the development of different chiral ligands for asymmetric CA reactions. Another major advancement occurred in 1997 when Feringa’s group developed the first catalytic, enantioselective tandem conjugate addition–aldol reaction of cyclic enones [19]. While tandem conjugate addition–α-functionalization reactions were well known prior to Feringa’s publication [20] (Scheme 1), this work was unique because of the high enantioselectivity that was observed through the use of a chiral ligand. Since this publication, many groups have developed methods to conduct tandem conjugate addition–alkylations, allylations and aldol reactions [21]. Through the work of Alexakis, Feringa and others, the range of Michael acceptors used in both CA and tandem CA–α-functionalization reactions have been expanded to include α,β-unsaturated ketones, aldehydes, lactones, esters, nitroolefins, and to a lesser extent α,β-unsaturated amides and lactams.

## Review

**Figure 1:**
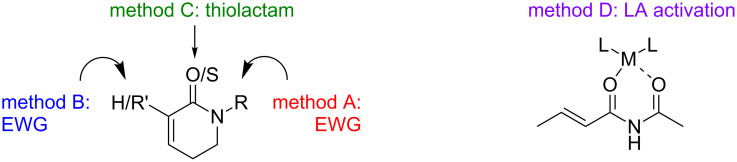
Methods to activate unsaturated amide/lactam systems.

The lack of development in the CA of α,β-unsaturated amides and lactams is not surprising because these substrates are significantly less reactive than other Michael acceptors. In order to improve the reactivity of amides and lactams, they have been modified in the following manner: A) placing an EWG on the nitrogen [22], B) placing an EWG on the α-carbon atom [23], C) converting the amide/lactam to a thioamide/lactam or D) activation by a Lewis acid [24] (Figure 1).

Method A is the most common method for activating the amide or lactam system. Method B has been generally employed in the synthesis of several natural products and pharmaceutical compounds [25]. When the starting substrate for the CA reaction is an unsaturated tertiary amide, the thioamide method is a useful strategy. Lewis acid activation (method D) has been used commonly for the 1,4-addition of stabilized nucleophiles, but there are some exceptions. The need to activate amides and lactams adds a level of difficulty in developing asymmetric CA (ACA) reactions.

Not surprisingly, a limited number of reviews focus on ACA reactions that are performed using α,β-unsaturated amides or lactams as Michael acceptors. In the many reviews that have been published on CA reactions, there are only a handful of examples provided where α,β-unsaturated amides or lactams are Michael acceptors. Since α,β-unsaturated amides and lactams are less reactive, the ACA reactions that have been developed for α,β-unsaturated ketones, esters and other substrates, would not necessarily work well on α,β-unsaturated amides and lactams. Therefore, the focus of this review is to highlight the work that has been done on the development of diastereoselective and enantioselective conjugate addition reactions using α,β-unsaturated amides and lactams. This is not an exhaustive account of all ACA reactions that employ α,β-unsaturated amides and lactams as Michael acceptors. Instead, the review aims to highlight significant work in this area. Though, the majority of the review will focus on examples where carbon nucleophiles are utilized; there are examples where heteronucleophiles are used throughout the review. There are only a couple of examples of sulfa-Michael additions that are mentioned in this review because this topic has recently been reviewed [26]. In terms of the mechanisms of the CA reactions, only those mechanisms that differ from the generally accepted ones will be discussed [4,27]. Finally, the terms conjugate addition, Michael addition and 1,4-addition will be used interchangeably throughout this review.

### 1 Diastereoselective conjugate additions (DCA)

Though CA reactions had been studied for decades, significant progress in the development of CA reactions using α,β-unsaturated amides and lactams was not observed until the 1980s [28–31]. Initial efforts toward performing the asymmetric CA reactions focused on the development of diastereoselective CA reactions. These reactions were achieved by performing conjugate additions to chiral Michael acceptors that blocked one face of the double bond from the incoming nucleophile, thus preferentially producing one diastereomer over the other. The chirality of the Michael acceptors can be attributed to one of two factors: 1) use of a chiral auxiliary attached to the nitrogen or 2) existing stereochemistry of the acceptor. In this section, we will briefly discuss the use of both types of chiral Michael acceptors in DCA of α,β-unsaturated amides and lactams.

#### 1.1 DCA using chiral auxiliaries

**Scheme 2:**
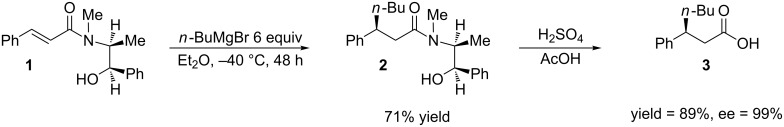
DCA of Grignard reagents to an L-ephedrine derived chiral α,β–unsaturated amide.

Chiral auxiliaries have been useful tools to induce stereoselectivity in C–C bond forming reactions [5,32–34]. Ideally, these compounds are stable, easily attached, removed and recoverable. One of the first examples of a DCA reaction using a chiral auxiliary was done by Mukaiyama and Iwasawa in 1981 [28]. DCA reactions were performed on a series of α,β-unsaturated amides, which employed L-ephedrine as a chiral auxiliary (Scheme 2).

Mechanistically, it was supposed that the Grignard reagent formed an internal chelate between the nitrogen and the oxygen on the auxiliary that locked the conformation of the molecule. The excess Grignard reagent would add to the double bond from the less sterically hindered side, thus leading to the diastereoselectivity. Note that when using Grignard reagents as nucleophiles in CA reactions, the possibility of the 1,2-addition of the carbonyl group is always a concern. In this case, the 1,2-addition product was not observed. The authors concluded that the 1,2-addition reaction did not occur because the magnesium salts that were present in the solution would be chelated strongly by the oxygen in the chiral auxiliary and the oxygen of the resulting enolate of the CA reaction. This chelation to the magnesium would retard the 1,2-addition reaction from occurring.

**Figure 2:**
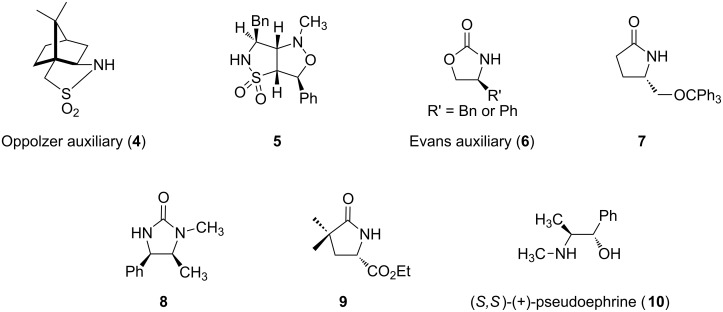
Chiral auxiliaries used in DCA reactions.

Since the Mukaiyama publication, there have been many chiral auxiliaries studied in DCA reactions of α,β-unsaturated amides [35]. Figure 2 highlights a few of the most common chiral auxiliaries that have been used.

**Scheme 3:**
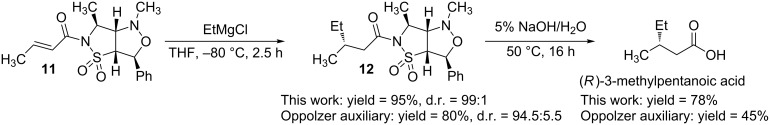
Comparison between auxiliary **5** and the Oppolzer auxiliary in a DCA reaction.

Like the Mukaiyama example, the Oppolzer auxiliary (**4**) was used in DCA reactions with Grignard reagents [36–37]. The advantages of using this auxiliary are that it is readily available from (−) or (+)-camphor-10-sulfonyl chloride, both the starting *N*-enoyl sultam and the product from the DCA reaction can be purified by recrystallization, and the auxiliary is easily removed. Another sultam-based chiral auxiliary that has been used is bicycle **5** [38]. The authors were able to demonstrate that use of auxiliary **5** in the DCA reaction of **11** provided an increase in diastereoselectivity and a higher yield of the product after deprotection of the sultam group over the Oppolzer auxiliary (Scheme 3).

**Scheme 4:**
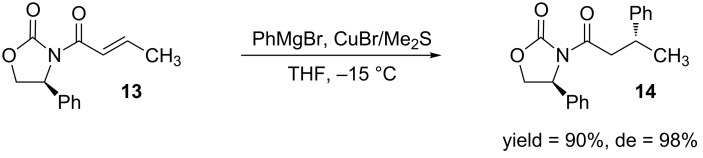
Use of Evans auxiliary in a DCA reaction.

The Evans auxiliary **6** has been used extensively in several asymmetric transformations such alkylations, allylations and most notably for aldol reactions [33,39]. It has also been utilized in DCA reactions of α,β-unsaturated amides [40–44]. One of the first examples was published by Hruby and co-workers, where they added Grignard reagents, in the presence of a CuBr–(CH_3_)_2_S complex, to a series of α,β-unsaturated-*N*-alkenoyl-2-oxazolidinones [43] (Scheme 4).

**Figure 3:**
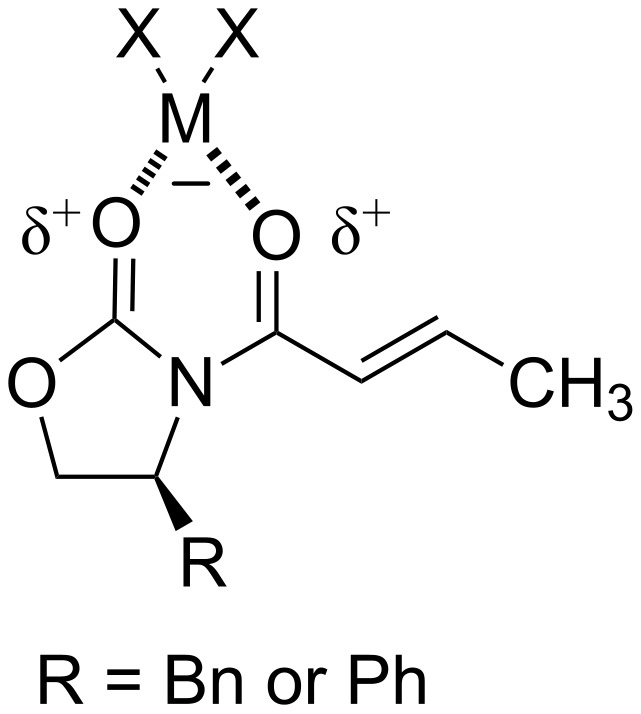
Lewis acid complex of the Evans auxiliary [43].

The diastereoselectivity of the reactions using the Evans auxiliary is attributed to the ability of the Lewis acidic metal to form a complex between the two carbonyl oxygens (Figure 3).

This Lewis acid complex locks the molecule into one conformation, thus facilitating the diastereoselectivity. Though the structure depicted in Figure 3 is considered the standard model for diastereoselectivity, there are examples where changes in the Lewis acid, nature of the organocuprate, or choice of the chiral auxiliary can show a reversal in the diastereoselectivity of DCA reactions [40,45]. Other auxiliaries such as trityloxymethyl-γ-butyrolactam (**7**) [45–46], (4*R*,5*S*)-1,5-dimethyl-4-phenyl-2-imidazolidionone (**8**) [47] and ethyl (*S*)-4,4-dimethylpyroglutamate (**9**) [48] have been used in DCA reactions with α,β-unsaturated amides, though to a lesser extent than the Evans auxiliary.

**Scheme 5:**
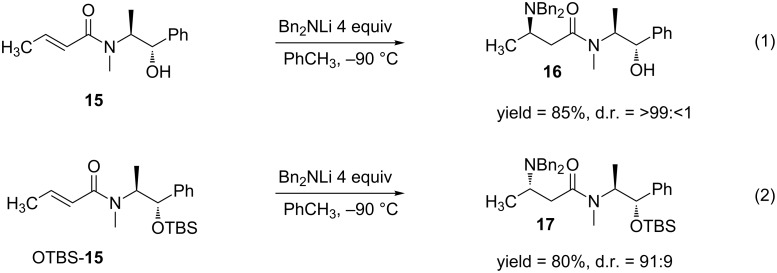
DCA reactions of α,β-unsaturated amides utilizing (*S*,*S*)-(+)-pseudoephedrine and the OTBS-derivative as chiral auxiliaries.

In recent years, (*S*,*S*)-(+)-pseudoephedrine has emerged as a chiral auxiliary for the use in DCA reactions of α,β-unsaturated amides [49–54]. Badía and co-workers published a report where they performed aza-Michael additions to various α,β-unsaturated amides attached to (*S*,*S*)-(+)-pseudoephedrine [51] (Scheme 5).

**Figure 4:**
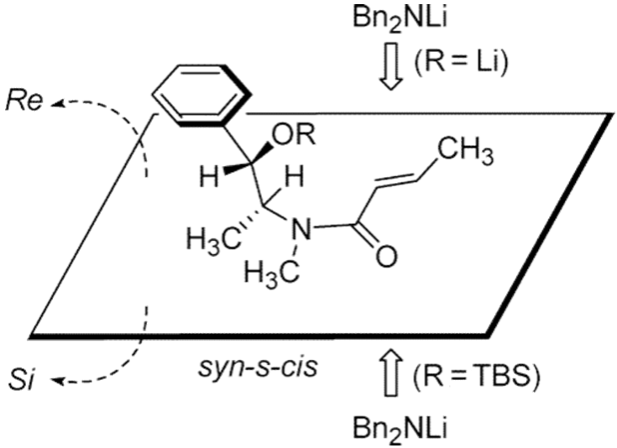
Proposed model accounting for the diastereoselectivity observed in the 1,4-addition of Bn_2_NLi to α,β-unsaturated amides attached to (*S,S*)-(+)-pseudoephedrine. Reprinted with permission from *J. Org. Chem.*, **2005**, *70*, 8790–8800. Copyright 2005 American Chemical Society.

Interestingly, when the oxygen in (*S*,*S*)-(+)-pseudoephedrine was protected, a reversal in diastereoselectivity was observed [54]. The authors attributed the difference in diastereoselectivity to the fact that when unmodified (*S*,*S*)-(+)-pseudoephedrine is used, a lithium alkoxide is produced in the presence of the lithium amide nucleophile. This alkoxide is believed to direct the addition of the lithium amide through coordination (Figure 4). On the other hand, when the oxygen in pseudoephedrine is TBS-protected (OTBS-**15**), this group sterically blocks one side of the double bond, thus the nucleophile attacks the opposite face.

**Scheme 6:**
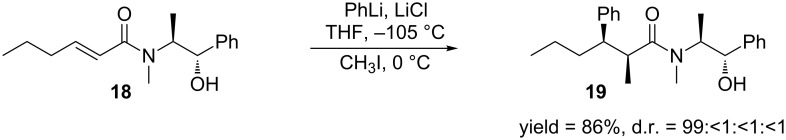
An example of a tandem conjugate addition–α-alkylation reaction of an α,β-unsaturated amide utilizing (*S*,*S*)-(+)-pseudoephedrine.

Prior to the development of the diastereoselective aza-Michael reactions, (*S*,*S*)-(+)-pseudoephedrine had been used as a chiral auxiliary for the diastereoselective α-alkylation of amides [55–57]. This literature precedent aided in the development of the tandem conjugate addition–α-alkylation reactions using (*S*,*S*)-(+)-pseudoephedrine as a chiral auxiliary [49–50] (Scheme 6).

These reactions proceeded with moderate to high yields (63–96%) and high diastereoselectivities. The authors noted that the diastereoselectivity of the initial CA reaction does not affect the stereochemical outcome of the alkylation step. In fact, the observed diastereoselectivity of the alkylation step is in agreement with previous reports on the diastereoselective α-alkylation of amides using (*S*,*S*)-(+)-pseudoephedrine as a chiral auxiliary [49–50,57].

**Scheme 7:**
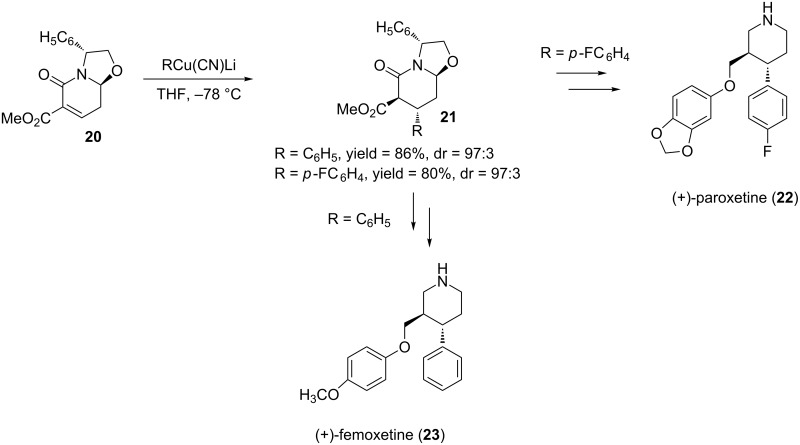
Conjugate addition to an α,β-unsaturated bicyclic lactam leading to (+)-paroxetine and (+)-femoxetine.

α,β-Unsaturated aminoalcohol-derived bicyclic lactams have been particularly useful in the synthesis of several natural products and pharmaceuticals [25,58–61]. Meyers and co-workers provided some of the first examples of DCA reactions utilizing these unsaturated lactams. They were able to obtain the 1,4-addition product in moderate to high yields and high diastereoselectivites [62–63]. Afterwards, Amat and co-workers utilized these unsaturated lactams in several DCA reactions. An example of these reactions is shown in Scheme 7 where the conjugate addition of **20** provided the corresponding product both in high yield and diastereoselectivity (97:3) [64]. Following a series of steps, the 1,4-addition products were converted to either (+)-paroxetine (R = *p*-FC_6_H_4_) or (+)-femoxetine (R = C_6_H_5_) (Scheme 7).

#### 1.2 DCA to inherently chiral Michael acceptors

When DCA reactions are performed using a chiral Michael acceptor, the diastereoselectivity is dictated by the overall stereochemistry of the Michael acceptor. The advantage of this approach over the use of a chiral auxiliary is that the addition and removal of a group to induce diastereoselectivity is not required, hence making this approach more atom economical. The disadvantage of performing DCA reactions using chiral substrates is that the overall stereochemistry of the Michael acceptor may either produce the undesired diastereomer in high yield or a low diastereoselectivity may be observed. Due to the complex nature of the chiral starting material, structural changes that would enhance the diastereoselectivity are typically difficult to execute. Despite this disadvantage, this approach has been utilized in the synthesis of several natural products [7–8,25,65–70]. The following section highlights a couple examples.

**Scheme 8:**
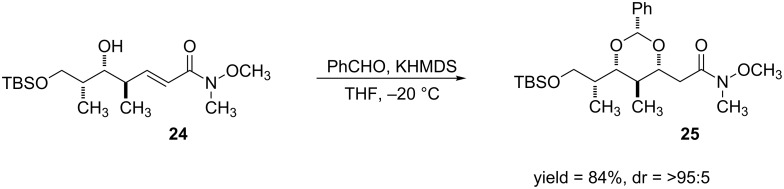
Intramolecular conjugate addition reaction to α,β-unsaturated amide.

In 1993, Evans and Gauchet-Prunet developed a method to synthesize protected syn-1,3-diols by performing intramolecular conjugate additions to a series of α,β-unsaturated esters and amides [71] (Scheme 8).

Upon treatment of **24** with KHMDS and benzyaldehyde, a hemiacetal forms which provides the alkoxide nucleophile for the DCA reaction. These reactions proceeded in good yields (71–84%) and high diastereoselectivities. This work has been useful because the syn-1,3-diol moiety is commonly observed in many natural products, most notably in polyol macrolide antibiotics [72–74].

**Scheme 9:**
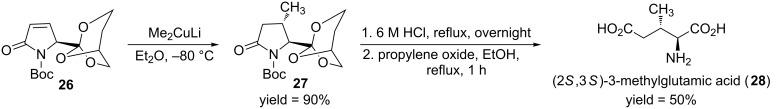
Conjugate addition to an α,β-unsaturated pyroglutamate derivative.

Performing CA reactions on chiral lactams has been a common method to access several natural products and unnatural amino acids [58,75–77]. For example, Oba and co-workers performed conjugate additions to an α,β-unsaturated pyroglutamate derivative where the carboxylic acid was protected as a 5-methyl-2,7,8-trioxabicyclo[3.2.1]octane (ABO ester) [66] (Scheme 9).

The authors were able to execute the conjugate addition of **26** using a Gilman reagent in high yield. The diastereoselectivity was not determined for the CA product. Instead, the authors converted the product to 3-methylglutamic acid **28** and they were able to observe only one diastereomer.

DCA reactions have been shown to be a useful method to perform asymmetric CA reactions. Initially, these reactions were done using chiral auxiliaries, which have provided the 1,4-addition product in high yield and diastereoselectivity. The use of chiral auxiliaries in DCA reactions has been useful because they can be easily attached and removed or transformed into a variety of functional groups. Though the utility of chiral auxiliaries in DCA reactions has been demonstrated in α,β-unsaturated amides, this method has not been extensively applied to α,β-unsaturated lactams. Another drawback to this approach is that a stoichiometric amount of the chiral auxiliary is required to induce diastereoselectivity. On the other hand, both α,β-unsaturated amides and lactams have been utilized as chiral Michael acceptors in DCA reactions. These reactions, which rely on the inherent stereochemical information of the Michael acceptor, have been useful in the synthesis of a number of natural products and pharmaceutical agents.

### 2 Enantioselective conjugate additions (ECA)

Enantioselective conjugate addition (ECA) reactions, especially catalytic variants, address the limitations of DCA reactions. ECA reactions do not require the addition and/or removal of groups on the Michael acceptor in order to achieve good stereoselectivity, which makes these reactions atom economical. Also, the low catalyst loading allows for the use of a variety of chiral ligands. Herein, various types of ECA reactions are discussed.

#### 2.1 Copper-catalyzed ECA reactions

During the early development of CA reactions, Grignard reagents were initially studied as potential nucleophiles for these reactions. Unfortunately, mixtures of the 1,4-addition and the acylation products were formed. Upon further investigation, it was discovered that the use of copper in CA reactions will preferentially form the 1,4-addition product [1]. Since then, copper has been the most commonly used metal in DCA and ECA reactions. In terms of the nucleophiles that are used, mainly alkyl organometallic reagents derived from zinc, aluminium, lithium and magnesium are used [1,3–4]. Alkenyl, alkynyl and aryl organometallic reagents have been used less frequently [78–84].

Feringa and co-workers reported the first copper-catalyzed ECA reactions of α,β-unsaturated lactams in 2004 [22]. They were able to perform these reactions in high yields and enantioselectivities by using a phosphoramide ligand [85]. Table 1 highlights the different substrates and alkyl metal nucleophiles that were studied in this work.

**Table 1:** Copper-catalyzed ECA reactions of alkyl nucleophiles to α,β-unsaturated lactams.

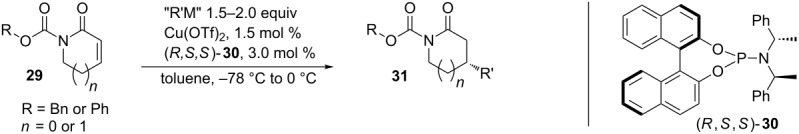					

Entry	R/*n*	“R’M”	Time (h)	Conv. (yield) %	ee (%)

1	Bn/1	Et_2_Zn	3	100 (70)	75
2	Ph/1	Et_2_Zn	2	100 (65)	95
3	Ph/1	Bu_2_Zn	4	100 (52)	>90
4	*t*-Bu/0	Et_2_Zn	4	90 (15)	35
5	Ph/1	Me_3_Al	2	100 (78)	68
6	Ph/1	Et_3_Al	1	100 (88)	28
7	*t*-Bu/0	Et_3_Al	2	95 (25)	3

Though the use of the Cbz group in the ECA reaction provided the product in good yield and enantioselectivity (Table 1, entry 1), entries 2 and 3 show that the best enantioselectivities were observed with the *N*-carboxyphenyl protecting group. The authors were also able to employ the more reactive alkylaluminium reagents in this reaction, although there was a decrease in the enantioselectivity (Table 1, entries 5 and 6). When the ECA of a 5-membered lactam was performed with an organozinc reagent, the reaction proceeded with high conversion, but the product was isolated in low yield and enantioselectivity (Table 1, entry 4). Alternatively, the authors tried to use the triethylaluminium reagent on the 5-membered lactam (Table 1, entry 7), but the enantioselectivity was worse than using diethylzinc. So far, this methodology is limited to using 6-membered lactams.

In 2013, Alexakis and co-workers reported the copper-catalyzed enantioselective 1,4-addition of a variety alkyl- and alkenylalanes to N-protected 5,6-dihydropyrrolinones [86]. In their report, they applied the same system that they developed for the asymmetric 1,4-addition of alkenylalanes to N-substituted-2,3-dehydro-4-piperidones [78]. It is important to note that addition of trimethylaluminium as a coactivator was necessary for these reactions to work [78,87] (Table 2).

**Table 2:** Copper-catalyzed ECA reactions of alkenyl nucleophiles to an α,β- unsaturated lactam.

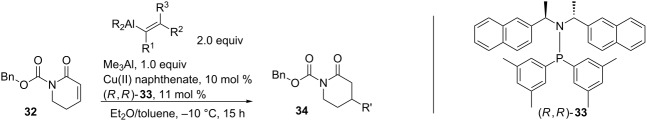					

Entry	R^1^	R^2^	R^3^	Yield (%)	ee (%)

1	H	*n*-Bu	H	53	86
2	H	*n*-Bu	H	70	86
3	H	Cy	H	54	82
4	H	*t*-Bu	H	66	72
5	H	(CH_2_)_3_Cl	H	58	84
6	H	Ph	H	69	90
7	Me	H	H	54	74
8	*n*-Bu	H	H	30	51
9	H	H	CH_2_O-*t*-Bu	47	12

Overall, the reactions in Table 2 show that moderate to good yields and good enantioselectivities can be achieved with a variety of alkenylalanes. Most notably, this methodology can be applied to the addition of functionalized alkenyl groups (Table 2, entry 5).

The authors also applied their methodology to the 1,4-addition of alkylalanes to an α,β-unsaturated lactam (Table 3).

**Table 3:** Copper-catalyzed ECA reactions of alkylalane nucleophiles to an α,β-unsaturated lactam.

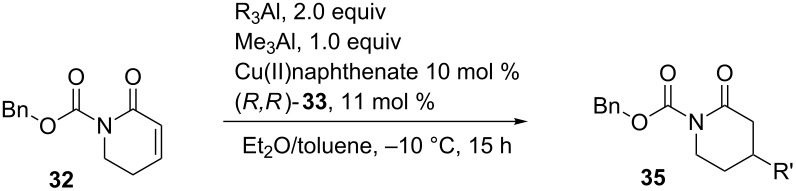			

Entry	R	Yield (%)	ee (%)

1	Me	54	94
2	Et	45	96
3	iBu	42	86
4	Ph	51	86

As compared to Feringa’s work with alkylalanes, yields of these reactions are lower, but the enantioselectivities are much higher. It is important to note that use of triethylaluminium in Alexakis’ work and diethylzinc in Feringa’s work provided the desired 1,4-addition product in comparable yield and enantioselectivity. Also, Alexakis and co-workers expanded the scope of this reaction by performing the 1,4-addition with triisobutylaluminium and triphenylaluminium (Table 3, entries 3 and 4), which provided the products in moderate yields and high enanitoselectivities.

So far, only copper-catalyzed ECA reactions of α,β-unsaturated lactams have been discussed. These reactions have also been applied to α,β-unsaturated *N*-alkenoyloxazolidinones. For instance, Hird and Hoveyda developed the copper-catalyzed asymmetric 1,4-addition of alkylzinc reagents to α,β-unsaturated *N*- alkenoyloxazolidinones [88]. The authors employed a chiral triamide phosphane in order to induce stereoselectivity, which is quite different from other chiral ligands that have been used in ECA reactions. The Hoveyda group has utilized amino acid-based ligands, which were rapidly identified through parallel screening methods [89–90], for ECA of a variety of Michael acceptors [91–94]. Table 4 shows a series of α,β-unsaturated *N*-alkenoyloxazolidinones that were used in the copper-catalyzed asymmetric 1,4-addition.

**Table 4:** Copper-catalyzed ECA of alkylzinc reagents to α,β-unsaturated *N*-alkenoyloxazolidinones.

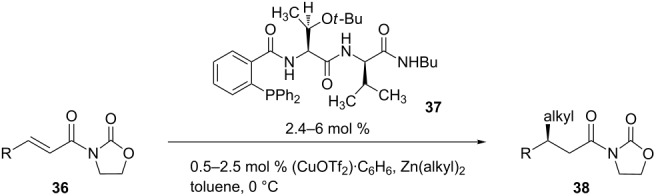							

Entry	R	Zn(alkyl)_2_	Mol % **37**	Mol % Cu salt	Time [h]	Yield (%)	ee (%)

1	Me	ZnEt_2_	2.4	1.0	1.3	95	95
2	Me	Zn[iPr(CH_2_)_3_]_2_	5.0	1.0	12	61	93
3	Me	Zn(iPr)_2_	2.4	0.5	15	95	76
4	*n*-Pr	ZnEt_2_	2.4	1.0	3	86	94
5	*n*-Pr	Zn[iPr(CH_2_)_3_]_2_	2.4	1.0	24	89	95
6	(CH_2_)_3_OTBS	ZnEt_2_	2.4	1.0	6	95	>98
7	iPr	ZnEt_2_	2.4	1.0	24	88	92

As shown in Table 4, these 1,4-addition reactions proceeded with good to excellent yields and enantioselectivities.

In 2006, Pineschi and co-workers reported the copper-catalyzed asymmetric 1,4-addition of alkylzinc reagents to acyclic α,β-unsaturated imides [95]. In this case, the authors used a phosphoramidite as the chiral ligand. They were also able to obtain the 1,4-addition product for α,β-unsaturated substrates that had an aryl group on the β-position, which was not achieved with the methodology that was developed by Hird and Hoveyda. Table 5 highlights selected examples of this reaction.

**Table 5:** Copper-catalyzed asymmetric 1,4-additions of alkylzinc reagents to acyclic α,β-unsaturated imides.

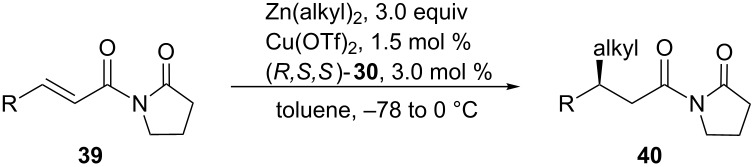				

Entry	R	Alkyl	Yield (%)	ee (%)

1	Pr	Et	80	84
2	Me	iPr	78	60
3	iPr	Et	75	95
4	Ph	Et	78	98
5	*p*-CF_3_C_6_H_4_	Et	84	>99
6	*p*-OMeC_6_H_4_	Et	74	94

The reaction proceeded in moderate to high yield and with good to high enantioselectivities.

**Scheme 10:**
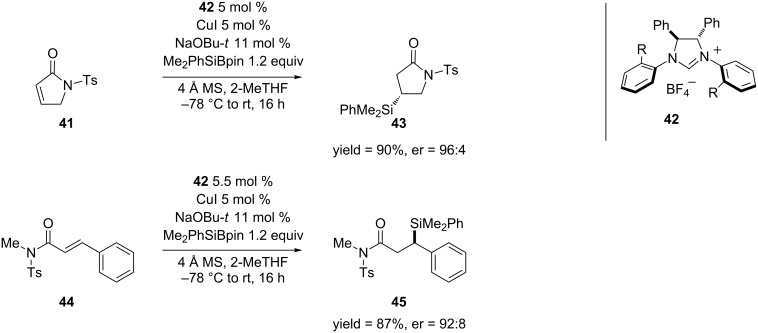
Cu(I)–NHC-catalyzed asymmetric silylation of α,β-unsaturated lactams and amides.

Though carbon nucleophiles are used in the majority of the copper-catalyzed ECA reactions of α,β-unsaturated amides and lactams, Procter and co-workers have expanded this methodology to the addition of silicon [96]. This reaction is particularly useful because the silyl group can be transformed into a number of functional groups [97]. In Procter’s work, they used a Cu(I)–NHC (N-heterocyclic carbene) system to catalyze the asymmetric transfer of silicon from PhMe_2_SiBpin [98–99] to α,β-unsaturated amides and lactams through activation of the Si–B bond [96] (Scheme 10).

Scheme 10 shows selected examples of the α,β-unsaturated lactams and amides that were used for the 1,4-silylation. Overall, the reactions were achieved in both high yields and enantioselectivities with a variety of substrates.

**Scheme 11:**
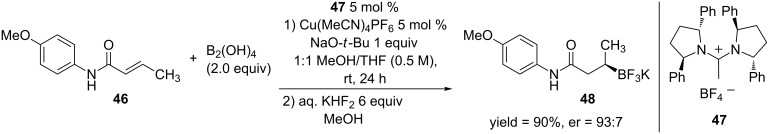
Asymmetric copper-catalyzed 1,4-borylation of an α,β-unsaturated amide.

In addition to silicon, much work has been done on the asymmetric 1,4-additon of boron to α,β-unsaturated carbonyl compounds. Enantioenriched organoboron reagents are useful because they can be used in cross-coupling reactions [98,100] or they can be converted into the corresponding alcohol [101] while retaining the stereochemical information. Transition metals such as Cu [102–112], Rh [113–115], Ni [116–117], Pd [116,118] and Pt [119–120] have all been successfully used to catalyze the 1,4-additon of boron to α,β-unsaturated carbonyl compounds. Of the transition metals mentioned, copper is the most cost-effective and least toxic choice. Though there are many examples of copper-catalyzed 1,4-additions of boron, most of the reports use bis(pinacolato)diboron (B_2_pin_2_) as a source of boron. In 2013, Molander and co-workers reported the asymmetric 1,4-addition of bisboronic acid (BBA) and tetrakis(dimethylamino)diboron to various α,β-unsaturated carbonyl compounds [121]. Use of bisboronic acid and tetrakis(dimethylamino)diboron provides boron sources that are more atom economical than B_2_pin_2_. Scheme 11 shows an example of the asymmetric 1,4-borylation where the authors used an α,β-unsaturated amide as the Michael acceptor.

**Scheme 12:**
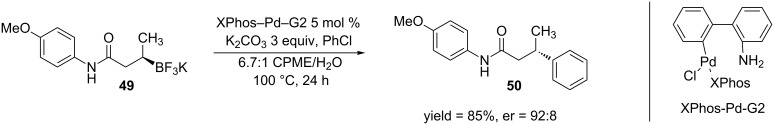
Asymmetric cross-coupling **49** to phenyl chloride.

The authors were able to obtain high yields and enantioselectivities for a variety of α,β-unsaturated carbonyl compounds. Also, they cross-coupled the β-borylated amides with various aryl and heteroaryl chlorides to yield the β-(hetero)arylated amides in high yields while maintaining stereochemical integrity. Scheme 12 shows an example of this cross-coupling reaction.

So far, there are not many examples of copper-catalyzed ECA reactions of α,β-unsaturated amides and lactams. One limitation to the methodology that has already been reported is that 1,4-addition of carbon nucleophiles to pyrrolinones have only resulted in low yields and enantioselectivities. Compared to the number of existing methods for copper-catalyzed ECA reactions of α,β-unsaturated ketones and other substrates, there is definitely a need for additional research in this area.

#### 2.2 Rhodium-catalyzed ECA reactions

Unlike copper-catalyzed CA reactions, the analogous rhodium-catalyzed reactions have only been developed in the last couple of decades. In 1997, Miyaura and co-workers reported the first examples of rhodium being used in 1,4-addition reactions. In this report, they used a rhodium(I) catalyst to perform 1,4-additions of aryl- and alkenylboronic acids to α,β-unsaturated ketones [122]. One year later, Hayashi and Miyaura reported the asymmetric variant of this reaction [123]. Since the publication of these seminal papers, rhodium has been used extensively in ECA reactions [124–135].

Rhodium-catalyzed ECA reactions have many advantages over the copper variant: (1) The reactions can be performed in protic or aqueous solvents. (2) Aryl-, alkenyl- and alkynyl nucleophiles are routinely used, where there are only limited examples in the copper-catalyzed version. (Note: In contrast, there have not been any rhodium-catalyzed CA reactions of alkyl nucleophiles.) Finally, (3) no 1,2-addition is observed in the rhodium variant because the organometallic reagents that are used are less reactive than the organolithium and -magnesium reagents used in the copper-catalyzed reactions [125].

**Scheme 13:**
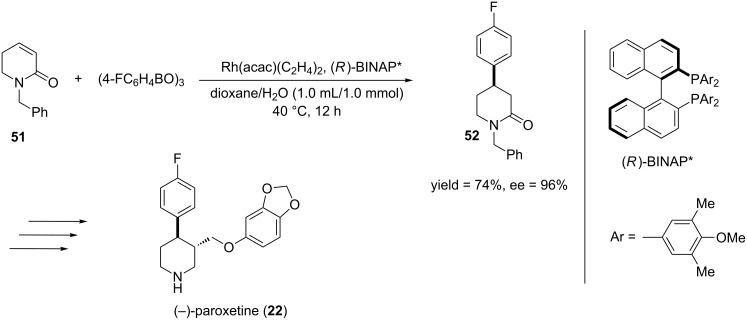
Rhodium-catalyzed asymmetric 1,4-arylation of an α,β-unsaturated lactam.

One of the first examples of a rhodium-catalyzed asymmetric 1,4-addition of lactams was reported by Hayashi and co-workers [136]. They reported that the key to achieving high yields for this reaction was to use arylboroxines instead of arylboronic acids and the addition of 1 equiv of water to boron. Also, highest yields were obtained when the reaction was run at 40 °C, which differs from reports where the reactions had to be run at 100 °C when other Michael acceptors were used [123,130,137–141]. In order to demonstrate the utility of this reaction, Hayashi and co-workers performed the rhodium-catalyzed ECA of **51** to yield 4-arylpiperidinone **52**. Compound **52** is a key intermediate in the synthesis of the pharmaceutical agent, (−)-paroxetine [142] (Scheme 13).

**Scheme 14:**
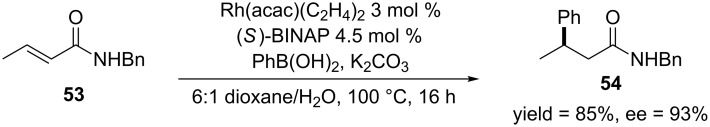
Rhodium-catalyzed asymmetric 1,4-arylation of an α,β-unsaturated amide.

In 2001, Sakuma and Miyaura reported the first rhodium-catalyzed asymmetric 1,4-addition of α,β-unsaturated amides [143]. They were able to obtain high enantioselectivities using a catalyst generated from Rh(acac)(C_2_H_4_) and (*S*)-BINAP. Unfortunately, the 1,4-addition products were only obtained in moderate yields because of incomplete conversion of the starting material. This problem could be rectified by the addition of an aqueous base. According to their discussion, the aqueous base converted the Rh(acac) complex to the corresponding RhOH complex. Such RhOH complexes have been proposed in the literature as active intermediates in the transmetallation of organoboronic acids [122,144–145]. Scheme 14 highlights an example of the rhodium-catalyzed asymmetric 1,4-addition of amides.

**Scheme 15:**
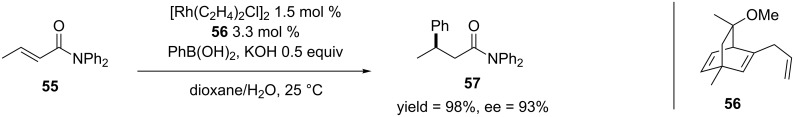
Rhodium-catalyzed asymmetric 1,4-arylation of an α,β-unsaturated amide using a chiral bicyclic diene.

In seminal reports for the rhodium-catalyzed ECA reactions, BINAP or a BINAP derivative was used as the chiral ligand for these reactions. Hayashi in 2003 [146] and Carreira in 2004 [147] demonstrated that chiral bicyclic dienes can act as useful chiral ligands for rhodium-catalyzed ECA reactions. In Carreira’s report, they examined the effectiveness of their diene ligand on the conjugate addition of an α,β-unsaturated amide (Scheme 15). They were able to obtain the 1,4-addition product both in high yield and enantioselectivity. Since the publication of these results, many different types of chiral dienes have been used in rhodium-catalyzed ECA reactions [148–149].

**Scheme 16:**
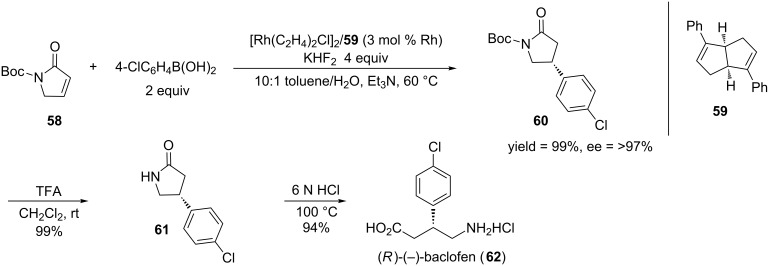
Synthesis of (*R*)-(−)-baclofen through a rhodium-catalyzed asymmetric 1,4-arylation of lactam **58**.

In 2011, Lin and co-workers reported the rhodium–diene-catalyzed asymmetric 1,4-arylation of γ-lactams [150]. Though He and co-workers previously reported a 1,4-arylation of γ-lactams, they obtained the product in moderate yields and good enantioselectivities [151]. When Lin and co-workers were developing their 1,4-arylation procedure, they decided to use a chiral diene because the literature had shown that these ligands provide higher reactivities and enantioselectivities than phosphine ligands in rhodium-catalyzed asymmetric reactions [152–153]. Scheme 16 shows the rhodium-catalyzed asymmetric 1,4-arylation that was developed by Lin and co-workers and the subsequent transformation to the GABA_B_ receptor agonist, (*R*)-(−)-baclofen.

**Scheme 17:**
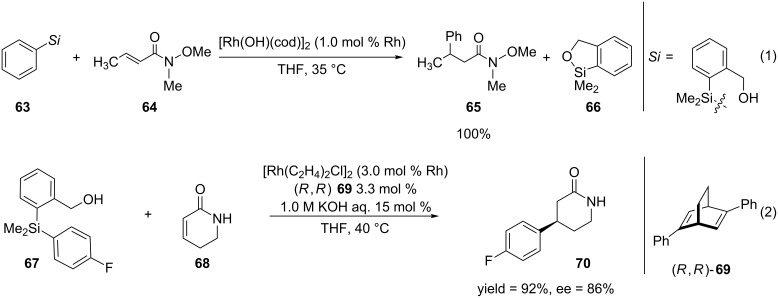
Rhodium-catalyzed asymmetric 1,4-arylation of an α,β-unsaturated amide and lactam employing organo[2-(hydroxymethyl)phenyl]dimethylsilanes.

Much of the work that has been discussed so far has employed the use arylboron reagents. In recent years, organosilicon reagents have emerged as key players in organic synthesis due to the their stability, low cost, and nontoxicity. Like organoboron reagents, organosilicon reagents are readily available, tolerant to functional groups, and easy to handle. Though organosilicon reagents have been used in rhodium-catalyzed asymmetric 1,4-additions, many of them rely on the use of moisture-sensitive organotri(alkoxy)silanes or the addition of an acid or base in order to obtain the product in high yield and enantioselectivity [154]. These observations led Hayashi, Hiyama and co-workers to develop mild conditions for the rhodium-catalyzed asymmetric 1,4-arylation and alkenylation [155]. They employed a series of organo[2-(hydroxymethyl)phenyl]dimethylsilanes [156–158] as organosilicon reagents and they also used these conditions for the 1,4-addition of α,β-unsaturated amides and lactams (Scheme 17).

Reaction 1 (Scheme 17) shows the rhodium-catalyzed 1,4-arylation of Weinreb amide **64** using phenyl[2-(hydroxymethyl)phenyl]dimethylsilanes. The authors were able to obtain the 1,4-addition product in quantitative yield in only 4 hours which is contrary to other reports of rhodium-catalyzed 1,4-arylation of α,β-unsaturated amides, where 20 hours were required for the completion of the reactions [130,154]. The authors expanded their methodology to an asymmetric version which is shown in the second reaction 2 of Scheme 17. Again the 1,4-addition product was obtained in high yield and enantioselectivity.

**Scheme 18:**
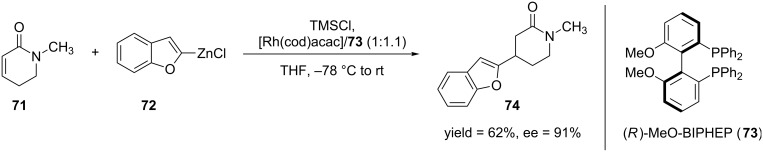
Rhodium-catalyzed asymmetric 1,4-arylation of an α,β-unsaturated lactam employing benzofuran-2-ylzinc.

Though both electron-rich and electron-poor aryl nucleophiles have been successfully added in rhodium-catalyzed asymmetric 1,4-arylations, heteroaryl nucleophiles have not been employed as extensively. Martin and co-workers expanded the scope of the rhodium-catalyzed asymmetric 1,4-addition by the development of conditions using 2-heteroaryltitanates and -zinc reagents [159]. With these conditions they obtained the 1,4-addition products in moderate to good yields and excellent enantioselectivities. The authors were also able to apply the methodology to an α,β-unsaturated lactam (Scheme 18).

**Figure 5:**
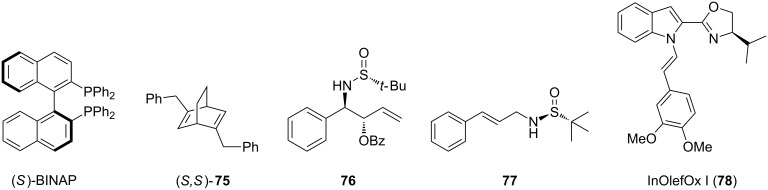
Further chiral ligands that have been used in rhodium-catalyzed 1,4-additions of α,β-unsaturated amides and lactams.

Though Binap and bicyclic diene ligands have been highlighted in this section, several other ligands have been used in rhodium-catalyzed asymmetric 1,4-additions of α,β-unsaturated amides and lactams (Figure 5).

Besides the previously referenced examples, (*S*)-Binap has also been used in the 1,4-addition of aryltrialkoxysilanes to a series of α,β-unsaturated amides [154]. In 2005, Hayashi and co-workers developed conditions for the 1,4-arylation of various Weinreb amides, which utilized (*S*,*S*)-**75** as the chiral ligand [130]. Xu and co-workers developed various *N*-sulfinyl homoallylic amines (**76**) [160] and *N*-cinnamyl sulfonamides (**77**) [161] ligands for the rhodium-catalyzed asymmetric 1,4-arylation of a variety of α,β-unsaturated carbonyl compounds. Finally, in 2012, Franzén and co-workers designed an indole-olefin-oxazoline (**78**) ligand for the 1,4-addition of α,β-unsaturated lactones and lactams [148]. With these ligands the desired products were obtained in moderate to excellent yields and good to excellent enantioselectivities.

This section illustrates that much work has been done in the development of asymmetric 1,4-arylations of α,β-unsaturated amides and lactams. The 1,4-arylation products were obtained in high yields and enantioselectivities through the use of a variety of chiral ligands and rhodium sources. Unfortunately, there are no examples of the rhodium-catalyzed ECA of alkenyl and alkynyl nucleophiles to α,β-unsaturated amides and lactams, which remains an area that is underdeveloped.

#### 2.3 Other transition-metal-catalyzed ECA reactions

**Scheme 19:**
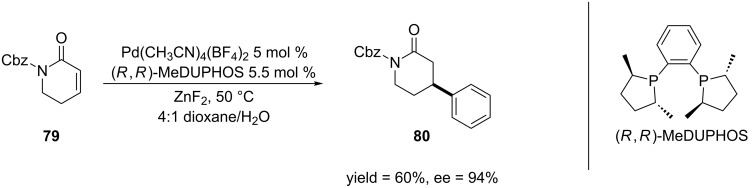
Palladium-catalyzed asymmetric 1,4-arylation of arylsiloxanes to a α,β-unsaturated lactam.

Though the majority of the reports on ECA of α,β-unsaturated amides and lactams utilize copper and rhodium as catalysts, other transition metals have also been used in these reactions. For example, Minnaard and co-workers developed the palladium-catalyzed asymmetric 1,4-arylation of arylsiloxanes to α,β-unsaturated carbonyl compounds [162]. The authors discovered that it was necessary to add a fluoride source to the reaction mixture in order to suppress side reactions, which has been reported by others to result in the isolation of the saturated carbonyl compound and phenol [163–166]. They were able to apply this methodology to the asymmetric 1,4-addition of lactam **79** to afford the 4-phenyl product in moderate yield and high enantioselectivity (Scheme 19).

**Scheme 20:**
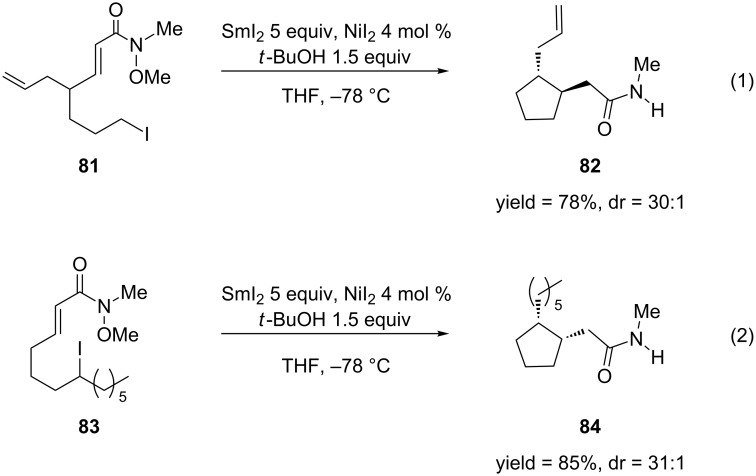
SmI_2_-mediated cyclization of α,β-unsaturated Weinreb amides.

Molander and Harris developed the samarium(II) iodide-mediated cyclization of α,β-unsaturated esters and amides [167] (Scheme 20). Previous methods for the cyclization of alkyl halides and α,β-unsaturated carbonyl systems include: (1) tin-mediated radical cyclization [168] or (2) nucleophilc cyclization via a carbanion generated through a lithium–halogen exchange [169–171]. The first method has limited utility because toxic byproducts are produced in the reaction that can be difficult to remove. The application of the second method is limited because the high reactivity of the lithium reagents restricts the functional groups that can be present in the starting material. The SmI_2_-mediated method provides a useful alternative because this reagent is mild and highly tolerant of functional groups.

Since it is well known that SmI_2_ causes the 1,4-reduction of α,β-unsaturated systems [172], nickel(II) iodide was added to retard this reaction by modifying the reduction potential of SmI_2_ [173–174]. The authors were pleased that the cyclization of Weinreb amides **81** and **83** proceeded in high yields and diasteroselectivities. As the authors used 5 equiv SmI_2_ in their reaction and it is well documented that SmI_2_ reduces N–O bonds [172,175], the 1,4-addition product was isolated as the amide, which resulted from the reduction of the *N*-OMe bond. Use of smaller quantities of SmI_2_ in the reaction produced only the reductive cleavage product (N–H) with no cyclization.

#### 2.4 Lewis acid-catalyzed ECA reactions

This section will focus on Lewis acid-catalyzed ECA reactions, which employ stabilized nucleophiles such as silyl enol ethers and amines. Chiral Lewis acids play a dual role in these reactions. They activate the Michael acceptors by coordinating with the carbonyl oxygen and they provide a chiral environment for the reaction to occur. The first part of this section will focus on the asymmetric 1,4-addition of carbon nucleophiles and the next two parts will discuss the addition of amines (aza-Michael addition) and phosphorous compounds.

**Figure 6:**
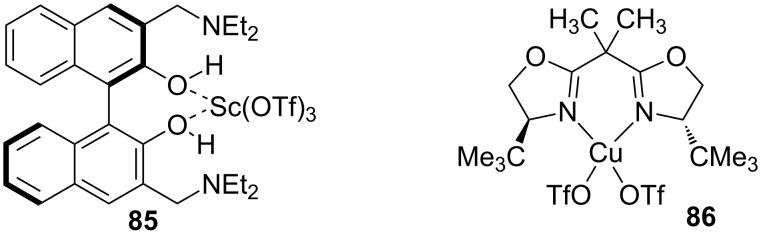
Chiral Lewis acid complexes used in the Mukaiyama–Michael addition of α,β-unsaturated amides.

**2.4.1 Asymmetric 1,4-addition of carbon nucleophiles:** Since its initial discovery [176–177], the Mukaiyama–Michael reaction has emerged as a reliable alternative to the use of metal enolates as nucleophiles in Michael reactions. Katsuki and co-workers reported the first application of this reaction to α,β-unsaturated amides [178]. Their reactions were promoted by either a Sc(OTf)_3_–3,3′-bis(diethylaminomethyl)-1,1′-bi-2-naphthol complex (**85**) or Cu(OTf)_2_–(*S*,*S*)-2,2′-isopropylidene-bis(4-*tert*-butyl-2-oxazoline) complex (**86**) (Figure 6).

**Scheme 21:**
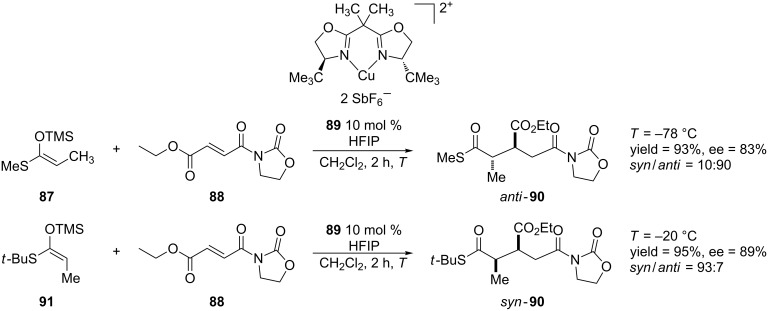
Mukaiyama–Michael addition of thioester silylketene acetal to α,β-unsaturated *N*-alkenoyloxazolidinones.

The latter bis(oxazoline) and its derivatives have become common chiral ligands in Lewis acid-mediated reactions [179]. The Evans group has developed several methods for Diels–Alder and aldol reactions that employed chiral bis(oxazoline) copper(II) complexes [180]. Starting in 1999, Evans and co-workers published a series of papers which reported the Mukaiyama–Michael addition to various α,β-unsaturated Michael acceptors [181–184]. Evans and co-workers also expanded their methodology for the development of asymmetric Mukaiyama–Michael addition of α,β-unsaturated *N*-acyloxazolidinones [182] (Scheme 21).

As shown in Scheme 21, these reactions proceed in high yields and enantioselectivities. Also, this scheme demonstrates that the geometry of the enolsilane drastically affects the diastereoselectivity of the reaction.

**Scheme 22:**
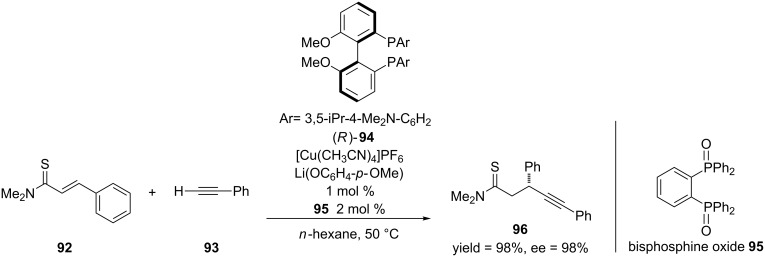
Asymmetric 1,4-addition of aryl acetylides to α,β-unsaturated thioamides.

Up to this point in this review, the use of alkynyl nucleophiles in the asymmetric 1,4-addition of α,β-unsaturated amides and lactams has not been discussed. Though alkynyl nucleophiles (in the form of metal alkynylides) have been used in both copper [185–187] and rhodium-catalyzed [188–191] 1,4-addition reactions, this methodology has not been expanded to the use of α,β-unsaturated amides and lactams as Michael acceptors. This is probably due to the combination of the low reactivity of these Michael acceptors and the low inherent nucleophilicity of the metal alkynylides. In 2010, Shibasaki and co-workers developed the asymmetric 1,4-addition of terminal alkynes to α,β-unsaturated thioamides [192]. In order to achieve this reaction, Shibasaki and co-workers used a soft Lewis acid/hard Brønsted base/hard Lewis base cooperative catalysis system, a strategy that has been used in other reactions [193–196] (Scheme 22).

**Scheme 23:**
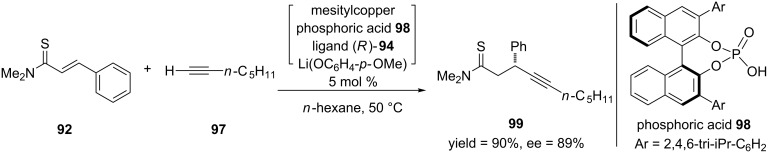
Asymmetric 1,4-addition of alkyl acetylides to α,β-unsaturated thioamides.

In the case of the reaction above, the alkynylide was generated from [Cu(CH_3_CN)_4_]PF_6_, (*R*)-3,5-iPr-4-Me_2_N-MeOBIPHEP, and Li(OC_6_H_4_-*p*-OMe) [197–198]. The hard Lewis base in this reaction was bisphosphine oxide **95**, which was added to enhance the Brønsted basicity of Li(OC_6_H_4_-*p*-OMe) by coordinating to the lithium counterion. The authors were able to obtain the 1,4-addition products in high yields and enantioselectivities when they used various aryl acetylides. Also, this methodology was expanded to various alkyl acetylides. In order to obtain high enantioselectivities, they varied the copper source, ligand and the hard Lewis base (Scheme 23).

**Scheme 24:**
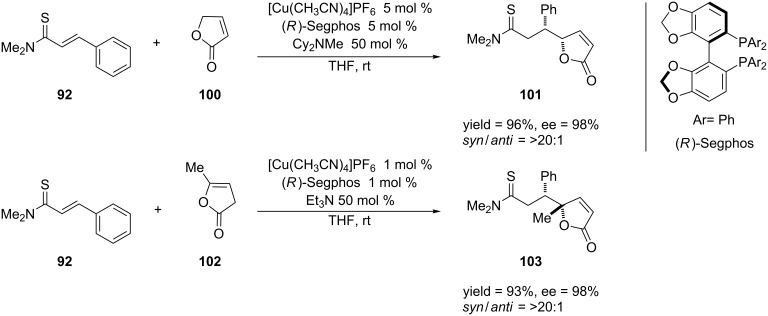
Asymmetric vinylogous conjugate additions of unsaturated butyrolactones to α,β-unsaturated thioamides.

The authors demonstrated the utility of this reaction by applying it towards the synthesis of a potent GPR40 agonist AMG 837 [199]. Shibasaki and co-workers expanded their application of soft Lewis acid/hard Brønsted base cooperative catalysis to the asymmetric vinylogous conjugate addition of α,β-unsaturated butyrolactones to α,β-unsaturated thioamides [200]. This reaction provides a method for producing enantioenriched butenolide units, which are found in many natural products [201–204] (Scheme 24).

The reactions in Scheme 24 demonstrate that the vinylogous conjugate addition of unsaturated butyrolactones to α,β-unsaturated thioamides proceeded in high yields, enantioselectivities and diastereoselectivities.

**Scheme 25:**
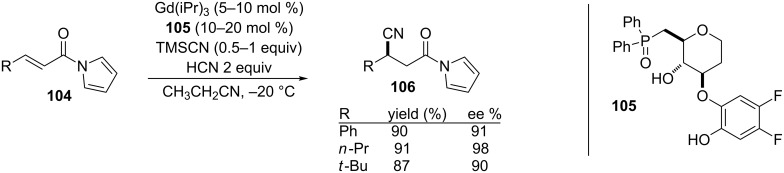
Gd-catalyzed asymmetric 1,4-cyanation of α,β-unsaturated *N*-acylpyrroles [205].

Starting in 2005, Shibasaki and co-workers reported both the gadolinium and strontium-catalyzed asymmetric 1,4-cyanation of α,β-unsaturated *N*-acylpyrroles [205–207]. *N*-Acylpyrroles have proven to be useful alternatives to Weinreb amides, because they share many of the same advantages but *N*-acylpyrroles are more reactive at the carbonyl unit and the tetrahedral intermediate is more stable than the Weinreb amides [208]. Based on their previous work on the Strecker reaction [209–211], Shibasaki and co-workers used a gadolinium-based catalyst for the 1,4-cyanation (Scheme 25).

Overall, this reaction proceeded in good to excellent yields and high enantioselectivies, with Michael acceptors having aryl or alkyl groups on the β-carbon.

**Scheme 26:**
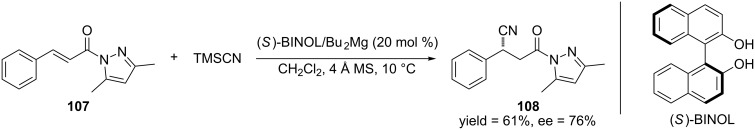
Lewis acid-catalyzed asymmetric 1,4-cyanation of α,β-unsaturated *N*-acylpyrazole **107**.

In 2014, Wang and co-workers reported a Lewis acid-catalyzed asymmetric 1,4-cyanation of α,β-unsaturated amides and ketones [212]. Though much work has been done in the development of 1,4-cyanations of α,β-unsaturated compounds [213–218], there are limited examples that use α,β-unsaturated amides as Michael acceptors [219]. Thus, the authors expanded the scope of this reaction by using a chiral Lewis acid as a catalyst. After screening many ligands, magnesium–BINOL complex was identified as suitable catalyst for the 1,4-cyanation of **107**. The authors obtained the 1,4-cyano products in both moderate to high yields and enantioselectivities for a variety of β-aryl substituted *N*-acylpyrazoles (Scheme 26).

**Scheme 27:**
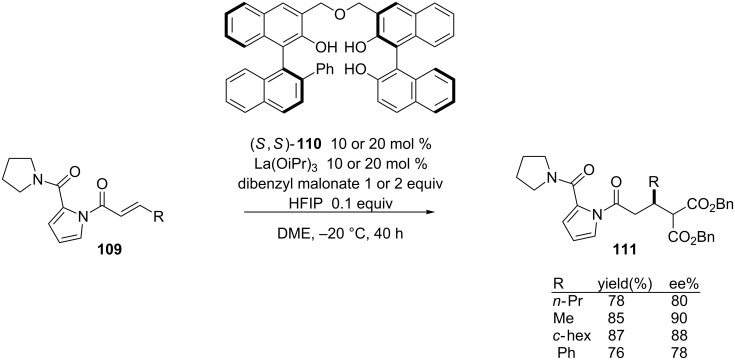
Lewis acid mediated 1,4-addition of dibenzyl malonate to α,β-unsaturated *N*-acylpyrroles.

In 2007, Shibasaki and co-workers reported the asymmetric 1,4-addition of dibenzyl malonate to α,β-unsaturated *N*-acylpyrroles catalyzed by a La(OiPr)_3_–linked BINOL complex [220]. Optimal yields and enantioselectivies were realized by adding 1,1,1,3,3,3-hexafluoroisopropanol (HIFP) to the reaction mixture and by placing steric bulk near the lanthanide metal. The authors were able to achieve the asymmetric 1,4-addition of dibenzyl malonate in good yields and enantioselectivies under these conditions (Scheme 27).

**Scheme 28:**
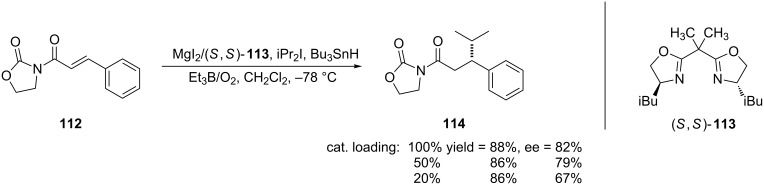
Chiral Lewis acid mediated 1,4-radical addition to α,β-unsaturated *N*-acyloxazolidinone [224].

Lewis acid catalysts have also been used to promote asymmetric 1,4-addition of radicals to α,β-unsaturated amides. Though there were a few previous reports of this reaction [221–223], Sibi and co-workers reported some of the first examples of the 1,4-radical addition to α,β-unsaturated *N*-alkenoyloxazolidinones using a substoichiometric amount of a chiral Lewis acid [224–225] (Scheme 28).

The authors were only able to decrease the catalyst loading to 20% in order to obtain the product in yields and enantioselectivities that were comparable to reactions that used a stoichiometric amount of the catalyst. Lowering the catalyst loading any further only led to longer reaction times and decreased yields.

**2.4.2 Asymmetric aza-Michael additions:** Aza-Michael additions have emerged as a powerful tool to form functionalized amines [226–228]. This reaction has been most commonly used to produce β-amino acids and their derivatives [229–230]. Not surprisingly, only a small amount of work in this area has utilized α,β-unsaturated amides or other carboxylic acid derivatives. This section will focus on the most significant findings that have been reported on the Lewis acid catalyzed aza-Michael additions of α,β-unsaturated amides and lactams [231–232].

**Scheme 29:**
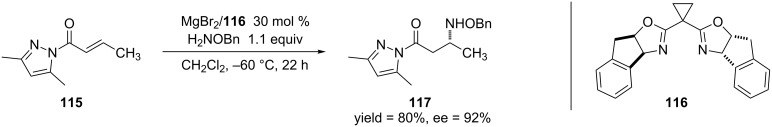
Aza-Michael addition of *O*-benzylhydroxylamine to an α,β-unsaturated *N*-acylpyrazole.

One of the first examples of an aza-Michael addition to α,β-unsaturated amides was reported by Sibi and co-workers [233]. In this work, they performed the 1,4-addition of *O*-benzylhydroxylamine to pyrazole-derived α,β-unsaturated amides using a chiral Lewis acid derived from bisoxazoline **116** (Scheme 29).

The authors obtained the 1,4-addition products in optimal yields and enantioselectivities when 30 mol % of the catalyst was used. Interestingly, when the Lewis acid is switched to the lanthanide triflate, Yb(OTf)_3_ or Y(OTf)_2_, the opposite configuration of the product was obtained (the *S* enantiomer was obtained vs. the *R* enantiomer that is obtained when MgBr_2_ is used).

**Scheme 30:**
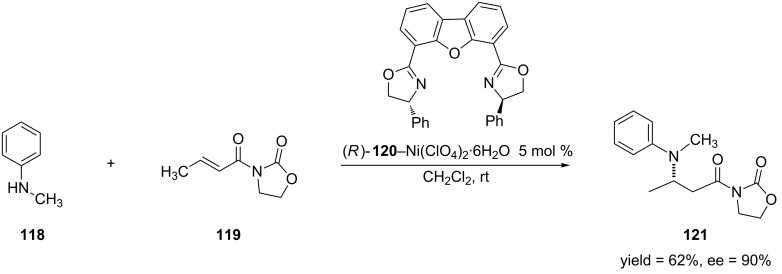
An example of the aza-Michael addition of secondary aryl amines to an α,β-unsaturated *N*-acyloxazolidinone.

In 2001, the Jørgensen group reported the first aza-Michael addition of secondary aryl amines [234]. The authors obtained the 1,4-addition products in moderate to high yields and enantioselectivities by using a nickel–DBFOX–Ph [235–236] catalyst (Scheme 30).

**Scheme 31:**
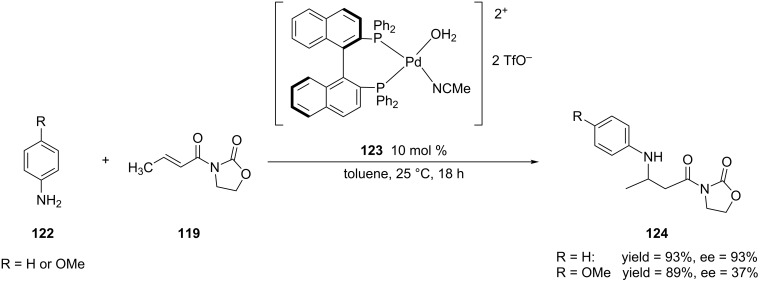
Aza-Michael additions of anilines to a α,β-unsaturated *N*-alkenoyloxazolidinone catalyzed by palladium(II) complex **123**.

Other chiral ligands have been used in addition to chiral bisoxazolines for asymmetric aza-Michael additions. For example, Hii and co-workers reported the first example of the use of a palladium(II) complex for the aza-Michael additions of selected α,β-unsaturated *N*-alkenoyloxazolidinones [237] (Scheme 31).

**Scheme 32:**
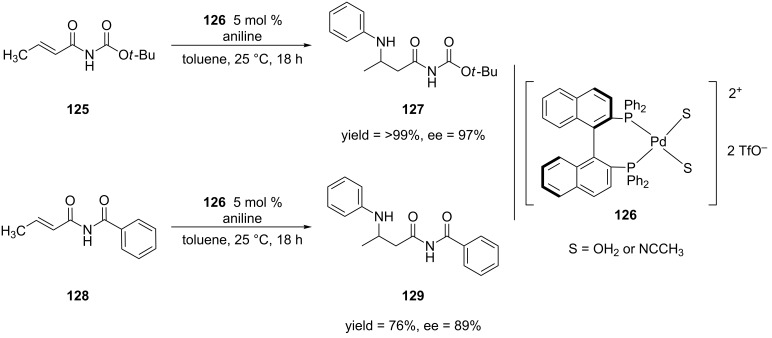
Aza-Michael additions of aniline to an α,β-unsaturated *N*-alkenoylbenzamide and *N*-alkenoylcarbamate catalyzed by palladium(II) complex **126**.

This reaction worked best when aniline was the aromatic amine that was used for the addition. Though the use of 4-Cl-C_6_H_4_NH_2_ gave similar results as with the parent compound aniline, the 1,4-addition reaction proceeded in high yield but low enantioselectivity when 4-OMe-C_6_H_4_NH_2_ was used as the nucleophile. This result revealed that these reaction conditions only work for electron-deficient anilines. Despite this limitation, Hii and co-workers expanded the methodology to other α,β-unsaturated *N*-alkenoylbenzamides [238] and carbamates [239–240] (Scheme 32).

**Scheme 33:**
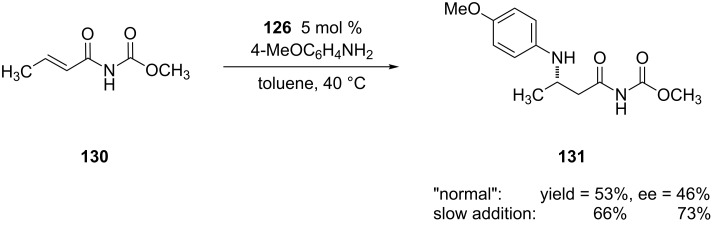
Difference between aza-Michael addition ran using the standard protocol versus the slow addition protocol.

In order to find means to overcome the low enantioselectivities observed when electron-rich anilines are used in their protocol for aza-Michael additions, Hii and co-workers performed a detailed mechanistic study of this reaction [241]. Based on this study, the rate-limiting step of the aza-Michael addition was the coordination of the catalyst to the 1,3-dicarbonyl moiety of the Michael acceptor. They were also able to determine that the aniline competitively binds to the catalyst. In order to avoid deactivation of the catalyst by the free aniline, the authors maintained a low aniline concentration during the reaction by using a syringe pump to add the aniline over 20 hours. Scheme 33 shows the difference in observed enantioselectivity of the aza-Michael addition using the previous protocol and the slow addition protocol.

**Scheme 34:**
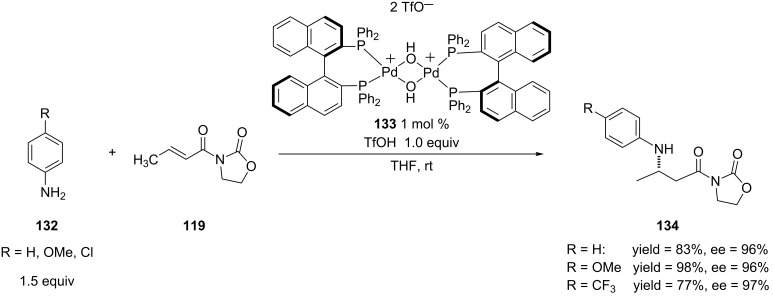
Aza-Michael additions of aryl amines salts to an α,β-unsaturated *N*-alkenoyloxazolidinone catalyzed by palladium(II) complex **133**.

In the report by Sodeoka and co-workers, the authors took a different approach to increasing the enantioselectivity of the 1,4-addition of electron-rich anilines [242]. To circumvent the issues raised by the Hii study [241], they added 1 equiv of triflic acid to 1.5 equiv of aniline to create the anilinium salt. The addition of the acid, along with the use of **133** as the chiral Lewis acid complex provided the 1,4-addition products in good to excellent yields and excellent enantioselectivities when both electron-deficient and -rich anilines were added (Scheme 34).

**Scheme 35:**
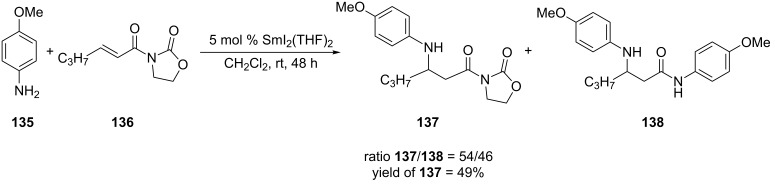
Aza-Michael addition of *N*-alkenoyloxazolidiniones catalyzed by samarium diiodide [244].

In 2008, Collin and co-workers developed the asymmetric aza-Michael addition of *N*-alkenoyloxazolidinones catalyzed by iodo(binaphtholato)samarium [243]. This group had previously reported the use of samarium diiodide in the 1,4-addition of aryl amines to *N*-alkenoyloxazolidinones [244]. In that report, they sometimes obtained a mixture of products, where one was the desired 1,4-addition product and the other product resulted from the 1,4-addition and acylation of the amine (Scheme 35). The side product was formed in higher amounts when electron-rich aryl amines were used.

**Scheme 36:**
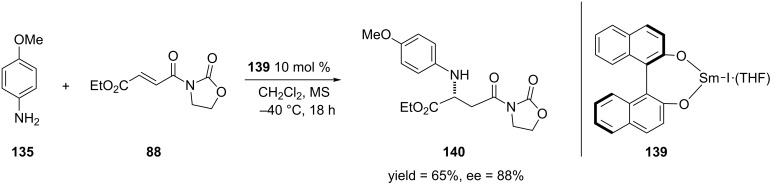
Asymmetric aza-Michael addition of *p*-anisidine to *α,β*-unsaturated *N*-alkenoyloxazolidinones catalyzed by iodido(binaphtholato)samarium [243].

In the asymmetric variant of this reaction, Collin and co-workers discovered that reducing the temperature to −40 °C diminished, and in some cases, eliminated the formation of the undesired product (Scheme 36). Overall, this iodo(binaphtholato)samarium-catalyzed asymmetric aza-Michael addition provided the 1,4-addition products in low to good yields and low to moderate enantioselectivities.

**Scheme 37:**
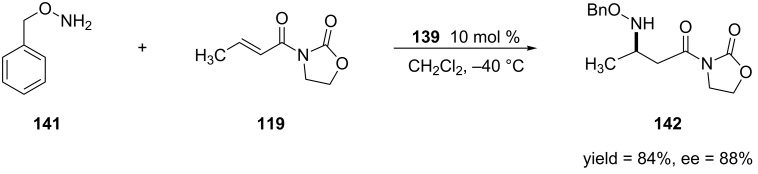
Asymmetric aza-Michael addition of *O*-benzylhydroxylamine to *N*-alkenoyloxazolidinones catalyzed by iodido(binaphtholato)samarium.

Collin and co-workers also expanded this methodology to the 1,4-addition of *O*-benzylhydroxylamine [245] (Scheme 37). Like the previous reactions, the formation of the undesired side product could be reduced by lowering the reaction temperature to −40 °C. Overall, the 1,4-addition products were produced in moderate to high yields and enantioselectivities.

**Scheme 38:**
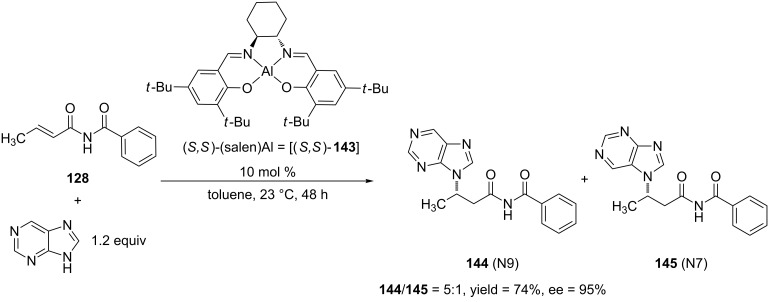
Asymmetric 1,4-addition of purine to an α,β-unsaturated *N*-alkenoylbenzamide catalyzed by (*S*,*S*)-(salen)Al.

Up to this point, aza-Michael additions, which use either anilines or *O*-benzylhydroxylamine, have been discussed. Gandelman and Jacobsen reported the first asymmetric 1,4-addition of various *N*-heterocycles to α,β-unsaturated imides [246]. This reaction represented a novel approach to synthesizing functionalized chiral *N*-heterocycles, which are studied in areas such as material science [247–248] and biological chemistry [249–252]. In the past, the Jacobsen group has used chiral (salen)Al complexes [253] as catalysts for the asymmetric 1,4-addition of azide [254], cyanide [219], substituted nitriles [255] and oximes [256] to α,β-unsaturated imides. The authors used these previous studies as the basis for the development of the 1,4-addition of *N*-heterocycles to α,β-unsaturated imides. They applied both substituted and unsubstituted purines, benzotriazole, and 5-substituted tetrazoles as nucleophiles to this reaction, which resulted in moderate to excellent yields and excellent enantioselectivities of the 1,4-addition products (Scheme 38).

As shown in Scheme 38, two 1,4-addition products were isolated because purine exists as a tautomeric mixture (N-7*H* and N-9*H*). Under the optimal reaction conditions, the N-9 regioisomer was produced preferentially.

**Scheme 39:**
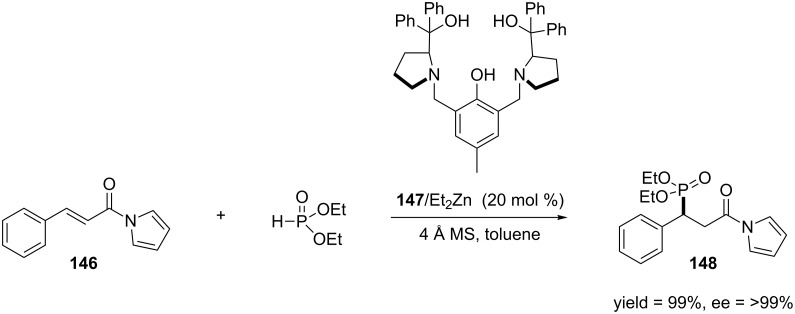
Asymmetric 1,4-addition of phosphites to α,β-unsaturated *N*-acylpyrroles.

**2.4.3 Asymmetric 1,4-addition of phosphorous compounds:** Optically active, phosphorous-containing compounds, most commonly phosphates, have been studied in the context of biology and medicine for decades. Due to the facile enzymatic cleavage of phosphates, phosphonates have been studied as phosphate mimics for many years [257–258]. Though the 1,4-addition of phosphites provides a direct route for accessing chiral phosphonates, there are only a few examples of this reaction [259–264]. In 2009, Wang and co-workers reported one of the first catalytic, enantioselective 1,4-addition of phosphites to α,β-unsaturated *N*-acylpyrroles [265] (Scheme 39).

**Scheme 40:**
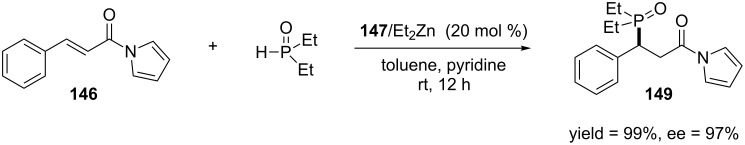
Asymmetric 1,4-addition of phosphine oxides to α,β-unsaturated *N*-acylpyrroles.

In their work, diethylzinc and **147** acts as a bifunctional catalyst system where the α,β-unsaturated system is activated through Lewis acid coordination and the catalyst acts as a directing group for the phosphite. Overall the reaction proceeds in both high yield and enantioselectivity. In 2010, Wang and co-workers expanded their methodology to the 1,4-addition of phosphine oxides. Similar to their previous work, this reaction provided the products in high yields and enantioselecitives [266] (Scheme 40).

#### 2.5 ECA employing organocatalysts

Organocatalysts have become powerful alternatives to perform reactions which have traditionally been carried out using metal or Lewis acid catalysts [267–268]. These reactions are simple to accomplish, and the catalysts are easy to handle and store. Also, many catalysts are commercially available or can be easily synthesized from commercially available materials. Organocatalysts can be placed into two catagories: 1) catalysts that covalently attach themselves to the starting materials (e.g., enamine [269] and iminium [270] catalysis) and 2) catalysts that interact with the starting materials through non-covalent interactions (e.g., hydrogen bonding [271]). In this section, the examples of ECA reaction of α,β-unsaturated amides and lactams will fit in the latter category.

**Scheme 41:**
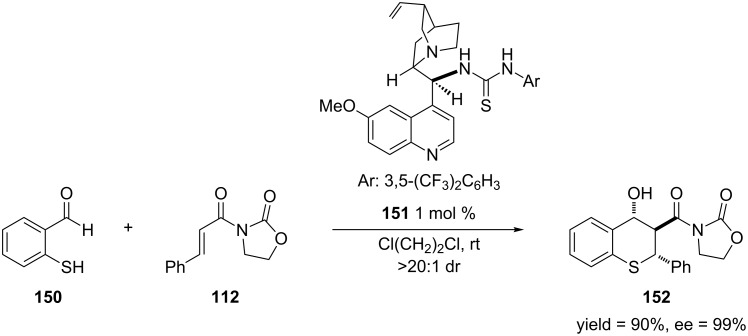
Tandem Michael-aldol reaction catalyzed by a hydrogen-bonding organocatalyst.

Wang and co-workers developed conditions to rapidly access stereochemically-enriched thiochromanes through a tandem Michael-aldol reaction of 2-mercaptobenzaldehydes and α,β-unsaturated imides [272]. This reaction was catalyzed by an organcatalyst that contains a thiourea group that is able to hydrogen bond with the imide moiety and a tertiary amine, which acts as an activating/directing group by hydrogen bonding to the thiol. Scheme 41 shows an example of this reaction. Overall, this reaction produced the thiochromenes in high yields, enantioselectivites and diastereoselectivities.

**Scheme 42:**
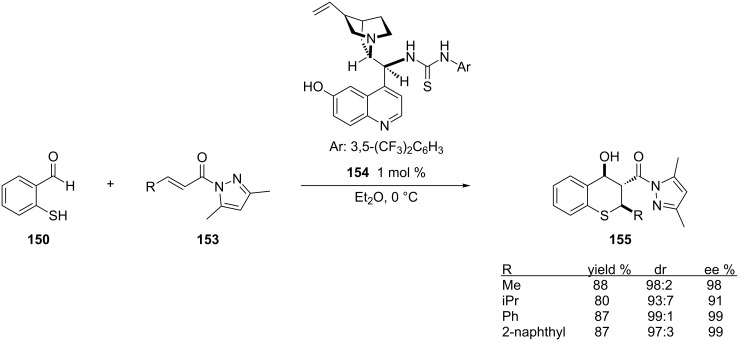
Examples of the sulfa-Michael–aldol reaction employing α,β-unsaturated *N*-acylpyrazoles.

One drawback of this work is that the authors only employed β-aryl-substituted α,β-unsaturated *N*-alkenoyloxazolidinones. Dong and co-workers expanded the scope of this reaction by using both β-aryl and alkyl-substituted Michael acceptors for the sulfa-Michael–aldol reaction [273]. Also, they replaced the oxazolidinone with a pyrazole on the Michael acceptor which led to obtaining the 1,4-addition product in high yields, enantioselectivities and diastereoselectivities (Scheme 42).

**Scheme 43:**
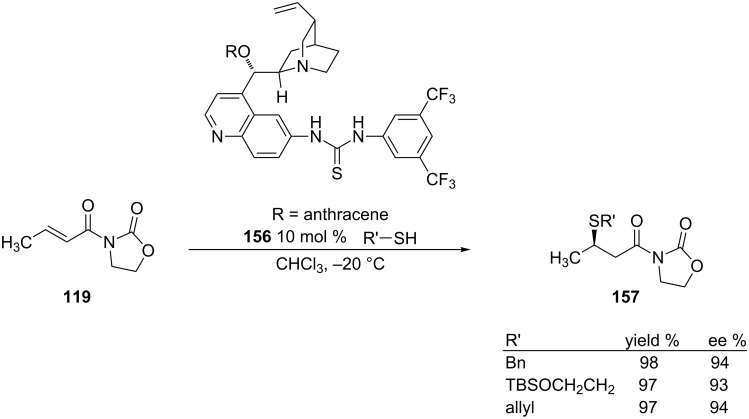
Example of the sulfa-Michael addition of α,β-unsaturated *N*-alkenoyloxazolidinones.

A couple years after Wang’s report on the tandem sulfa-Michael–aldol reactions, Deng and co-workers reported the use of a cinchona alkaloid catalyst in the simple asymmetric 1,4-addition of thiols to α,β-unsaturated *N*-alkenoyloxazolidinones [274] (Scheme 43).

**Figure 7:**
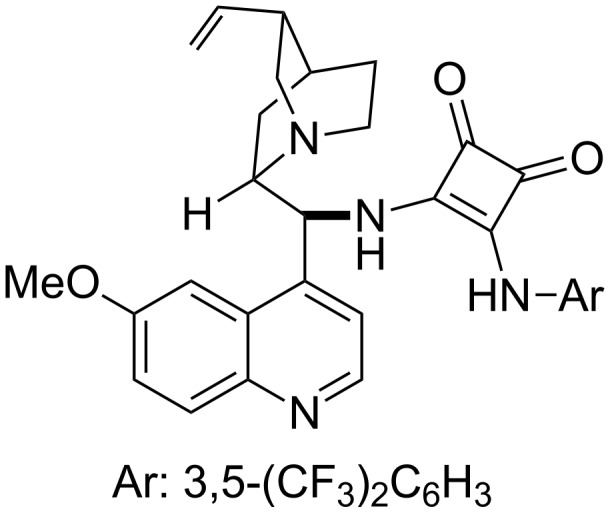
Structure of cinchona alkaloid-based squaramide catalyst.

Under these conditions, the authors obtained the 1,4-addition products in excellent yields and enantioselectivities. In 2011, Chen and co-workers applied their cinchona alkaloid-based squaramide catalyst to the asymmetric sulfa-Michael addition of α,β-unsaturated *N*-alkenoyloxazolidinones [275] (Figure 7). Unlike the previous example in Scheme 43, products were obtained in excellent yields and enantioselectivies while these reactions were carried out at room temperature. Also, the scope of the thiols used was expanded to include various alkyl derivatives.

For the past few years, Hoveyda and co-workers have reported on the development of a metal-free, NHC-catalyzed method for the 1,4-addition of boron to carbonyl compounds [276–277]. In 2012, this group reported a catalytic, enantioselective version of this reaction, which included the use of a variety of α,β-unsaturated carbonyl compounds [278]. They included the 1,4-borylation of α,β-unsaturated Weinreb amides. Overall, these reactions proceeded in good to excellent yields and excellent enantioselectivies (Table 6).

**Table 6:** Enantioselective 1,4-borylation of α,β-unsaturated Weinreb amides.

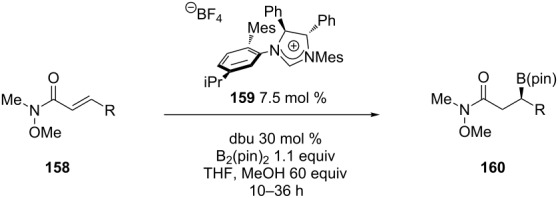					

Entry	R	Temp (°C)	Conv. (%)	Yield (%)	er

1	Ph	50	86	61	86.5:13.5
2	*p*-BrC_6_H_4_	50	>98	70	94:6
3	*n*-pentyl	50	77	74	95:5
4	(CH_2_)_2_OTBS	66	96	92	93:7
5	iBu	66	78	73	95:5
6	iPr	50	67	60	95:5

**Scheme 44:**
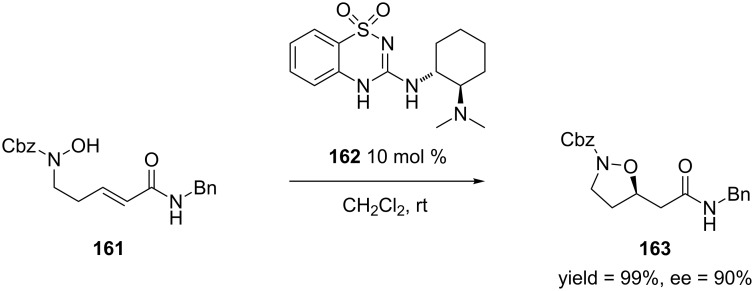
Asymmetric intramolecular oxa-Michael addition of an α,β-unsaturated amide.

In 2013, Takemoto and co-workers developed an organocatalyst-mediated asymmetric intramolecular oxa-Michael addition of α,β-unsaturated esters and amides [279]. Though oxa-Michael additions of α,β-unsaturated esters and amides have been reported in the literature [280], Takemoto’s work provides mild conditions for the reaction, which will minimize unwanted side reactions in the presence of a base. Scheme 44 shows an example of this reaction.

**Scheme 45:**
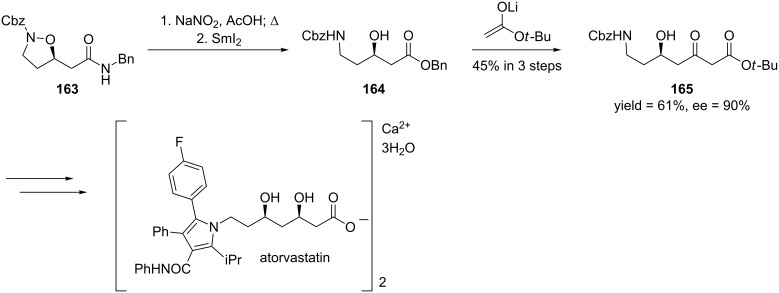
Formal synthesis atorvastatin.

The authors generally obtained the oxa-Michael addition products in good to high yields and excellent enantioselectivities. In order to demonstrate the utility of this reaction, the authors used isoxazolidine **163** in the formal synthesis of atorvastatin [281–283] (Scheme 45).

Isoxazolidine **163** was converted to the δ-amino-β-hydroxyester **164** after transforming the benzyl amide to the benzyl ester and cleaving the N–O bond. Then, compound **164** was converted to the β-keto ester **165** through a cross-Claisen condensation. Ester **165** can be converted to atorvastatin after a series of steps [284].

## Conclusion

Asymmetric conjugate addition reactions have become powerful tools for the formation of stereochemically enriched C–C, C–heteroatom bonds. The utility of these reactions have been demonstrated through their use in the synthesis of numerous natural products and pharmaceutical agents. The last couple of decades have shown a significant increase in the development of asymmetric conjugate addition reactions, especially in the catalytic, enantioselective variants of these reactions. Unfortunately, much of this work does not include the use of α,β-unsaturated amides and lactams due to their inherently low reactivity. This review highlights the work of authors who were able to overcome this low reactivity in order to develop diastereoselective and enantioselective conjugate additions of α,β-unsaturated amides and lactams.

Although ACA reactions using α,β-unsaturated amides and lactams have been underdeveloped in general, much progress has been made in the last two decades. One of the most significant developments occurred in the mid-2000s when Feringa and Hoveyda/Pineschi reported some of the first methods for the catalytic, enantioselective 1,4-addtion of alkyl nucleophiles. This represented a significant breakthrough because these reactions had previous been well developed for other α,β-unsaturated compounds. Also, the use of a variety of carbon nucleophiles such as alkenyl, alkynyl and aryl nucleophiles and heteronucleophiles such as amines, silanes and alcohols have been reported. Often these reactions afford the product in high yields and enantioselectivities, thus providing valuable tools to access β-substituted carboxylic acid derivatives.

While progress has been made in the development of ACA reactions utilizing α,β-unsaturated amides and lactams, there is still work that needs to be done. For example, rhodium-catalyzed asymmetric 1,4-additions of alkenes and alkynes to α,β-unsaturated ketones and other substrates have been well established, yet these reactions have not been applied to α,β-unsaturated amides and lactams. Another area for development is the limited utilization of 5-membered lactams in ACA reactions. The 1,4-addition products of these substrates can easily be converted to unnatural amino acids and other key intermediates in the synthesis of a natural product or pharmacological agent. Thus there is still a need to continue making progress in the development of asymmetric 1,4-addition reactions that use α,β-unsaturated amides and lactams as Michael acceptors.
